# Measured and estimated data of non-linear BRAN channels using HOS in 4G wireless communications

**DOI:** 10.1016/j.dib.2018.02.005

**Published:** 2018-02-10

**Authors:** Mohammed Zidane, Said Safi, Mohamed Sabri

**Affiliations:** a Department of Physics, Faculty of Sciences and Techniques, Sultan Moulay Slimane University, Morocco; b Department of Mathematics and Informatics, Polydisciplinary Faculty, Sultan Moulay Slimane University, Morocco

**Keywords:** HOS, Non-linear quadratic systems, BRAN data, Blind identification, Blind equalization, ZF, MMSE, BER

## Abstract

The aim of this research is to develop a non-linear blind estimator able to represents a Broadband Radio Access Networks (BRAN) channels. In the one hand, we have used Higher Order Statistics (HOS) theory to build our algorithm. Indeed, we develop a non-linear method based only on fourth order cumulants for identifying the diagonal parameters of quadratic systems. In the other hand, the developed approach is applied to estimate the experimental channels, BRAN A, C and E data normalized for MC-CDMA, in non-linear case. However, the estimated data will be used in the blind equalization. The simulation results in noisy environment and for different signal to noise ratio (SNR) show the accuracy of develop estimator blindly (i.e., without any information about the input) with non-Gaussian signal input. Furthermore, in part of blind equalization problem the obtained results, using Zero forcing (ZF) and Minimum Mean Square Error (MMSE) equalizers, demonstrate that the proposed algorithm is very adequate to correct channel distortion in term the Bit Error Rate (BER). Finally, these estimated data present a necessary asset for conducting validation experiments, and can be also used as a baseline.

## 1 Specifications table

**Table:** 

Subject area	Signal processing and digital telecommunications
More Specific subject area	Blind non-linear channels identification, Blind equalization of MC-CDMA systems
Type of data	Tables
How data was acquired	Compensate the fading channel in term the BER in 4G Wireless Communications
Data Format	ETSI BRAN Mobile Channels
Data accessibility	Data is within this article

## 2 Value of the data

• Exploiting the HOS theory to develop a blind algorithm able to estimate non-linear real channels without reference to the measure;
• The estimated data provides information about the efficiencies of develop method;
• An analysis of the influence of the noise to estimated data of BRAN channels;
• The estimated data can be also used as a baseline;
• Exploiting the estimation data of BRAN channels in blind equalization;
• Can be used for wireless communications in order to compensate the fading channel in term the BER in 4G MC-CDMA systems.

### 2.1 Data

Three models, BRAN A, C and BRAN E, [1], [2] are used in this investigation. These models correspond to typical large open space indoor and outdoor environments with large delay spread. The data presented in Table 1, Table 2, Table 3 represent the delay and magnitudes of 18 targets of BRAN A, C and E channels respectively.

## 3 Experimental design, materials and methods

### 3.1 Non-linear channels representation: A problem formulation

The BRAN channel is modeled as the output of a non-linear quadratic system that is excited by an non-Gaussian signal input and is corrupted at its output by an additive Gaussian noise.

**Fig. 1:**
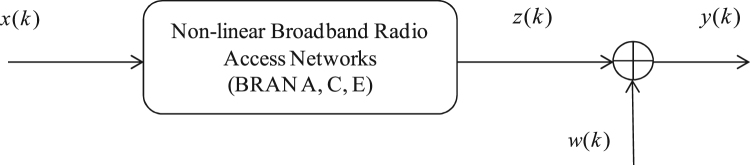
Non linear quadratic systems.

This system can be represented as follows (Fig. 1):

The output of this model is described by the following relationships:

(1) $$y(k)=\overset{q}{\underset{i=0}{\sum }}h(i,i)x^{2}(k-i)+w(k)$$

For this system we assume that:

• The considered noise sequence w(k) is assumed to be zero mean, independent and identically distributed (i.i.d), Gaussian, independent of x(k) with unknown variance;
• The order *q* is known. Unknown quadratic kernels include {h(i,i)∀i=1,…,q};
• The input sequence x(k) is (i.i.d) zero mean, stationary, non-Gaussian and with:
  $$C_{n,x}(\tau_{1},\tau_{2},\ldots ,\tau_{n-1})=\{\begin{array}{cc} \gamma_{n,x}, & \tau_{1}=\ldots =\tau_{n-1}=0;\\ 0 & \text{otherwise} \end{array}$$
  where $\gamma_{n,x}$ denotes the $n^{\mathrm{th}}$ order cumulants of the input signal x(k) at origin, with $\gamma_{2,x}=E[x^{2}(k)]\neq 0$, $\gamma_{3,x}=E[x^{3}(k)]\;\neq \;0$, $\gamma_{4,x}=E[x^{4}(k)]\;\neq \;0$, $\gamma_{6,x}=E[x^{6}(k)]\;\neq \;0$, $\gamma_{8,x}=E[x^{8}(k)]\;\neq \;0$;
• The system is supposed causal and truncated, i.e. h(i,i)=0 if i<0 and i>q with h(0,0)=1;
• The system is supposed stable, i.e. |h(i,i)|<∞.

In this section, we give the relationship linking the higher order statistics or cumulants with the diagonal parameters of quadratic systems.

The second and third order cumulant of the process {z(k)} are described by the following expressions respectively [3]:

(2) $$C_{2,z}(\tau )=(\gamma_{4,x}-\gamma_{2,x}^{2})\overset{q}{\underset{i=0}{\sum }}h(i,i)h(i+\tau ,i+\tau )$$

(3) $$C_{3,z}(\tau_{1},\tau_{2})=(\gamma_{6,x}-3\gamma_{2,x}\gamma_{4,x}+2\gamma_{2,x}^{3})\overset{q}{\underset{i=0}{\sum }}h(i,i)h(i+\tau_{1},i+\tau_{1})h(i+\tau_{2},i+\tau_{2})$$

The fourth order cumulants of the process {z(k)} is defined by [4]:

(4) $$\begin{array}{c} C_{4,z}(\tau_{1},\tau_{2},\tau_{3})=(\gamma_{8,x}\;-\;4\gamma_{6,x}\gamma_{2,x}\;-\;3\gamma_{4,x}^{2}+12\gamma_{4,x}\gamma_{2,x}^{2}\;-\;6\gamma_{2,x}^{4})\sum_{i=0}^{q}h(i,i)\times \;h(i\;+\;\tau_{1},i\;+\;\tau_{1})h(i\;+\;\tau_{2},i\;+\;\tau_{2})h(i\;+\;\tau_{3},i\;+\;\tau_{3}) \end{array}$$

### 3.2 Proposed non-linear blind estimator

In this subsection we develop a blind method for identifying non-linear BRAN channels and downlink MC-CDMA equalization.

The Fourier transform of Eqs. (2), (4) gives us respectively:

(5) $$S_{2,z}(\omega )=\eta_{2}H(-\omega ,-\omega )H(\omega ,\omega ),$$

where $\eta_{2}=(\gamma_{4,x}\;-\;\gamma_{2,x}^{2})$.

(6) $$S_{4,z}(\omega_{1},\omega_{2},\omega_{3})=\eta_{4}H(-\omega_{1}-\omega_{2}-\omega_{3},-\omega_{1}-\omega_{2}-\omega_{3})H(\omega_{1},\omega_{1})H(\omega_{2},\omega_{2})H(\omega_{3},\omega_{3}),$$

where $\eta_{4}=(\gamma_{8,x}\;-\;4\gamma_{6,x}\gamma_{2,x}\;-\;3\gamma_{4,x}^{2}+12\gamma_{4,x}\gamma_{2,x}^{2}\;-\;6\gamma_{2,x}^{4})$.

If we suppose that $\omega =\omega_{1}+\omega_{2}+\omega_{3}$, Eq. (5) becomes:

(7) $$S_{2,z}(\omega_{1}+\omega_{2}+\omega_{3})=\eta_{2}H(-\omega_{1}-\omega_{2}-\omega_{3},-\omega_{1}-\omega_{2}-\omega_{3})H(\omega_{1}+\omega_{2}+\omega_{3},\omega_{1}+\omega_{2}+\omega_{3})$$

Then, from Eqs. (6), (7) we obtain the following equation:

(8) $$S_{4,z}(\omega_{1},\omega_{2},\omega_{3})H(\omega_{1}+\omega_{2}+\omega_{3},\omega_{1}+\omega_{2}+\omega_{3})=\mu_{\left(4,2\right)}H(\omega_{1},\omega_{1})H(\omega_{2},\omega_{2})H(\omega_{3},\omega_{3})S_{2,z}(\omega_{1}+\omega_{2}+\omega_{3}),$$

where $\mu_{\left(4,2\right)}=\frac{\eta_{4}}{\eta_{2}}=\frac{\gamma_{8,x}-4\gamma_{6,x}\gamma_{2,x}-3\gamma_{4,x}^{2}+12\gamma_{4,x}\gamma_{2,x}^{2}-6\gamma_{2,x}^{4}}{\gamma_{4,x}-\gamma_{2,x}^{2}}$.

The inverse Fourier transform of Eq. (8) demonstrates that the fourth order cumulants, the autocorrelation function (second order cumulants) and diagonal parameters of quadratic systems, h(i,i), are combined by the following equation:

(9) $$\overset{q}{\underset{i=0}{\sum }}C_{4,z}(\tau_{1}\;-\;i,\tau_{2}\;-\;i,\tau_{3}\;-\;i)h(i,i)=\mu_{\left(4,2\right)}\overset{q}{\underset{i=0}{\sum }}h(i,i)h(\tau_{2}\;-\;\tau_{1}+i,\tau_{2}\;-\;\tau_{1}+i)\times \;h(\tau_{3}\;-\;\tau_{1}+i,\tau_{3}\;-\;\tau_{1}+i)C_{2,z}(\tau_{1}\;-\;i)$$

If we use the autocorrelation function property of the stationary process such as $C_{2,z}(\tau )\neq 0$ only for −q≤τ≤q and vanishes elsewhere if we take $\tau_{1}=2q$, Eq. (9) takes the form:

(10) $$\overset{q}{\underset{i=0}{\sum }}C_{4,z}(2q\;-\;i,\tau_{2}\;-\;i,\tau_{3}\;-\;i)h(i,i)=\mu_{\left(4,2\right)}h(q,q)h(\tau_{2}\;-\;q,\tau_{2}\;-\;q)h(\tau_{3}\;-\;q,\tau_{3}\;-\;q)C_{2,z}(q),$$

else if we suppose that $\tau_{2}=q$ the Eq. (10) becomes:

(11) $$\overset{q}{\underset{i=0}{\sum }}C_{4,z}(2q\;-\;i,q\;-\;i,\tau_{3}\;-\;i)h(i,i)=\mu_{\left(4,2\right)}h(q,q)h(\tau_{3}\;-\;q,\tau_{3}\;-\;q)C_{2,z}(q),$$

where, h(0,0)=1.

To simplify the (11), we consider the Eq. (4), and we take $\tau_{1}=\tau_{2}=q$ and $\tau_{3}=0$ thus:

(12) $$\;C_{4,z}(q,q,0)=(\gamma_{8,x}\;-\;4\gamma_{6,x}\gamma_{2,x}\;-\;3\gamma_{4,x}^{2}+12\gamma_{4,x}\gamma_{2,x}^{2}\;-\;6\gamma_{2,x}^{4})h^{2}(q,q),$$

and we use the Eq. (2) with $\tau_{1}=q$, (2) reduces:

(13) $$\;C_{2,z}(q)=(\gamma_{4,x}\;-\;\gamma_{2,x}^{2})h(q,q)$$

From (12), (13) we obtain:

(14) $$h(q,q)=\frac{\gamma_{4,x}\;-\;\gamma_{2,x}^{2}}{\gamma_{8,x}\;-\;4\gamma_{6,x}\gamma_{2,x}\;-\;3\gamma_{4,x}^{2}+12\gamma_{4,x}\gamma_{2,x}^{2}\;-\;6\gamma_{2,x}^{4}}\frac{C_{4,z}(q,q,0)}{C_{2,z}(q)}$$

Thus, we based on (14) for eliminating h(q,q) in (11), we obtain the following equation:

(15) $$\overset{q}{\underset{i=0}{\sum }}C_{4,z}(2q\;-\;i,q\;-\;i,\tau_{3}\;-\;i)h(i,i)=C_{4,z}(q,q,0)h(\tau_{3}\;-\;q,\tau_{3}\;-\;q),$$

where, $q\leq \tau_{3}\leq 2q$ according the hypothesis four in Section 2.

The system of (15) can be written under the matrix form as follows:

(16) $$\left(\begin{array}{ccc} C_{4,z}(2q-1,q-1,q-1) & \ldots & C_{4,z}(q,0,0)\\ C_{4,z}(2q-1,q-1,q)-\zeta & \ldots & C_{4,z}(q,0,1)\\ . & . & .\\ . & \;. & .\\ . & \;. & .\\ C_{4,z}(2q-1,q-1,2q-1) & \ldots & C_{4,z}(q,0,q)-\zeta \end{array}\right)\times \left(\begin{array}{c} h(1,1)\\ .\\ .\\ .\\ h(i,i)\\ .\\ .\\ .\\ h(q,q) \end{array}\right)=\left(\begin{array}{c} \zeta \;-\;C_{4,z}(2q,q,q)\\ -C_{4,z}(2q,q,q+1)\\ .\\ .\\ .\\ .\\ .\\ -C_{4,z}(2q,q,2q) \end{array}\right),$$

where $\zeta =C_{4,z}(q,q,0)$.

Or in more compact form, (16) can be written as follows:

(17) $$M_{q}h_{q}=d_{q},$$

with $M_{q}$ the matrix of size (q+1,q) elements, $h_{q}$ a column vector of size (q,1) and $d_{q}$ is a column vector of size (q+1,1). The least square solution of the system of Eq. (17) is given by:

(18) h^q=(MqTMq)−1MqTdq

## 4 Overview an MC-CDMA systems

In the purpose to support the anticipated multi-media intensive applications, we have needed a high data rate, this we pushed to use the future 4G communication systems. However, the demand for high data rates in 4G systems causes the transmitted signals to be subjected to frequency-selective fading. Indeed, Orthogonal Frequency Division Multiplexing (OFDM) and MC-CDMA are multicarrier modulation schemes that have been proposed for 4G systems due to their ability to achieve high spectral efficiency by using minimally spaced orthogonal subcarriers and provide robustness against frequency selectivity in wireless channels, without increasing the transceiver complexity [6].

The MC-CDMA signal can be generated by a inverse Fourier transform (IFFT) performed on the spreading code chips. Thus, the choice of spreading codes is fundamental [5], [7], [8]. Indeed, the complex symbol $a_{i}$ of each user *i* is, firstly, multiplied by each chip $c_{i,k}$ of Walsh-Hadamard spreading code, and then applied to the modulator of multicarriers. Each subcarrier transmits an element of information multiply by a code chip of that subcarrier. Under the hypothesis of $L_{c}$ equal to $N_{p}$, the expression of the signal transmitted at the output of the modulator is given by the following equation:

(19) $$x=\frac{1}{\sqrt{N_{p}}}\mathrm{Ca},$$

where, the matrix C represent the spreading codes:

(20) $$C=[c_{0},c_{1},\ldots ,c_{N_{u}-1}]$$

where, $c_{i}={\left[c_{i,0},c_{i,1},\ldots ,c_{i,L_{c}-1}\right]}^{T}$.

When $N_{u}$ users are active, the multi-user downlink MC-CDMA signal received at the input of the receiver, denoted by r(t), is given by the following expression:

(21) $$r(t)=\frac{1}{\sqrt{N_{p}}}\overset{P-1}{\underset{p=0}{\sum }}\overset{N_{p}-1}{\underset{k=0}{\sum }}\overset{N_{u}-1}{\underset{i=0}{\sum }}R\left\{\beta_{p}e^{j\theta_{p}}a_{i}c_{i,k}e^{2j\pi (f_{0}+k/T_{c})(t-\tau_{p})}\right\}+n(t)$$

After the equalization operation, the expression of the signal $s_{k}$ is given in vector form by the following expression:

(22) $$\begin{array}{c} s\;=\;\mathrm{Gr}=\;\mathrm{GHCa}\;+\;\mathrm{Gn}, \end{array}$$

where, $H=\mathrm{diag}\;[h_{0},h_{1},\ldots ,h_{N_{p}-1}]$ represents the complex channel frequency response.

The matrix $G=\mathrm{diag}\;[g_{0},\ldots ,g_{N_{p}-1}]$ represent the diagonal matrix composed of the coefficients $g_{k}$ equalization.

Or, in scalar form by the following expression:

(23) $$s_{k}=g_{k}h_{k}\left(\overset{N_{u}-1}{\underset{i=0}{\sum }}c_{i,k}a_{i}\right)+g_{k}n_{k}$$

After despreading and threshold detection, the data symbol of the user detected corresponds to the sign of the scalar produced between the vector of the received equalized signals, *s*, and the user-specific spreading code *i*, $c_{i}^{T}$, that is:

(24) ai^=<ciT,s>=∑k=0Np−1ci,ksk

Using Eqs. (23), (24) the general expression of the symbol detected for *i* user is given by the following equation:

(25) ai^=∑q=0Nu−1∑k=0Np−1ci,k(gkhkcq,kaq+gknk)=∑k=0Np−1ci,k2gkhkai︸U(i=q)+∑q=0Nu−1∑k=0Np−1ci,kcq,kgkhkaq︸M(i≠q)+∑k=0Np−1ci,kgknk︸N,

where the term *U*, *M* and *N* of Eq. (25) are, respectively, the signal of the considered user, a signals of the others users (multiple access interferences) and the noise pondered by the equalization coefficient and by spreading code of the chip.

If we suppose that the spreading code are orthogonal, i.e.,

(26) $$c_{i}^{T}c_{q}=\overset{N_{p}-1}{\underset{k=0}{\sum }}c_{i,k}c_{q,k}=0\;\forall \;i\;\neq \;q$$

Eq. (25) will become:

(27) ai^=∑k=0Np−1ci,k2gkhkai︸U+∑k=0Np−1ci,kgknk︸N

### 4.1 ZF equalizer

The goal of ZF minimize the peak distortion of the equalized channel, it is applying of the inverse of channel to the received signal and restores signal, defined as:

(28) $$g_{k}=\frac{1}{h_{k}}$$

The estimated received symbol, ai^ of symbol $a_{i}$ of the user *i* is described by:

(29) ai^=∑k=0Np−1ci,k2ai+∑k=0Np−1ci,k1hknk

The goal of the equalization is to extract $a_{i}$.

### 4.2 MMSE equalizer

The minimization of the function $E[|\varepsilon {|}^{2}]=E[|x_{k}-g_{k}r_{k}{|}^{2}]$, gives us the optimal equalizer coefficient. The equalization coefficients based on this MMSE criterion applied independently per carrier are equal to:

(30) $$g_{k}=\frac{h_{k}^{*}}{|h_{k}{|}^{2}+\frac{1}{\zeta_{k}}},$$

where $\zeta_{k}=\frac{E[|x_{k}h_{k}{|}^{2}]}{E[|n_{k}{|}^{2}]}$.

The estimated received symbol, ai^ of symbol $a_{i}$ of the user *i* is described by:

(31) ai^=∑k=0Np−1ci,k2|hk|2|hk|2+1ζkai+∑k=0Np−1ci,khk*|hk|2+1ζknk

## 5 Numerical simulations results

The work presented in this paper is structured around two neighboring themes. Identification of BRAN channels in the one hand, and downlink MC-CDMA equalization in the other hand. In the part of identification impulse response parameters of BRAN channels using HOS method we use the non-Gaussian input, and the additive noise is Gaussian, with symmetric distribution, zero mean, with the $m^{\mathrm{th}}$ order cumulants vanishes for m>2. Hence the utility to use the higher order cumulants domain. In the part of equalization problem of MC-CDMA systems we use the BPSK symbol constellation.

**Fig. 2:**
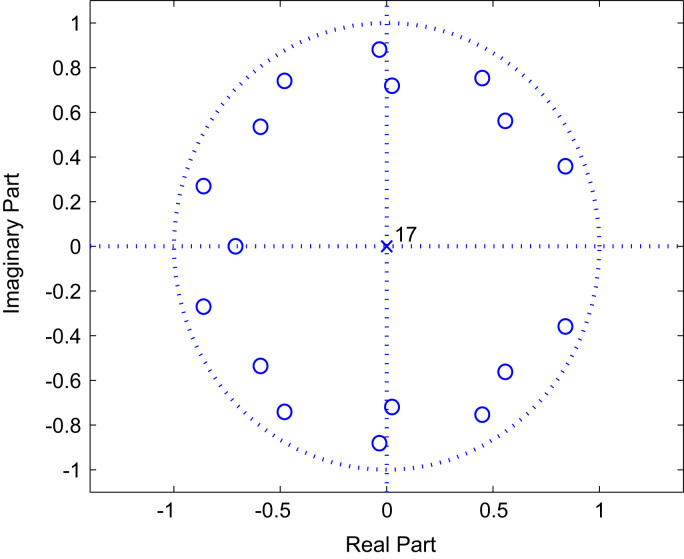
The zeros of Model BRAN A.

In this section, we present the numerical simulations results of the estimated data of BRAN A, C and E. The simulation is performed with MATLAB software in noise environment. To measure the strength of noise, we define the SNR by the following relationship:

(32) $$\mathrm{SNR}=10\;\log_{10}\left[\frac{\sigma_{z}^{2}(k)}{\sigma_{w}^{2}(k)}\right]$$

To measure the accuracy of the diagonal parameter estimation with respect to the real values, we define the Mean Square Error (MSE) for each run as:

(33) MSE=1q∑i=0q[h(i,i)−h^(i,i)h(i,i)]2,

where h^(i,i) and h(i,i), i=1,…,q, are respectively the estimated and the real parameters in each run.

**Fig. 3:**
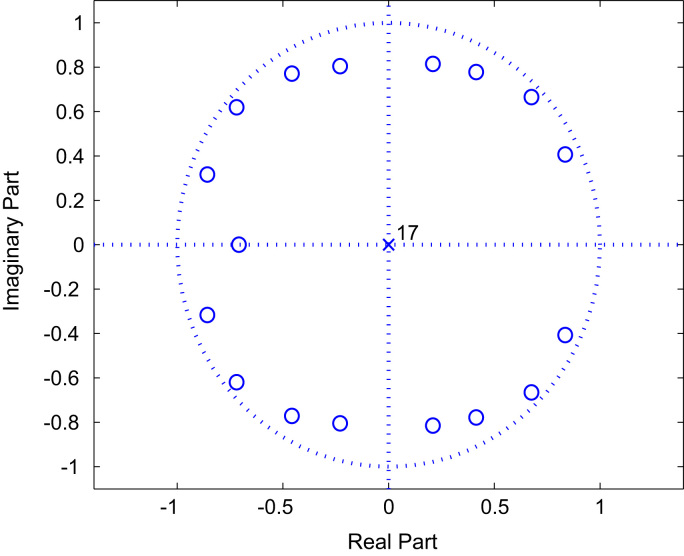
The zeros of Model BRAN C.

**Fig. 4:**
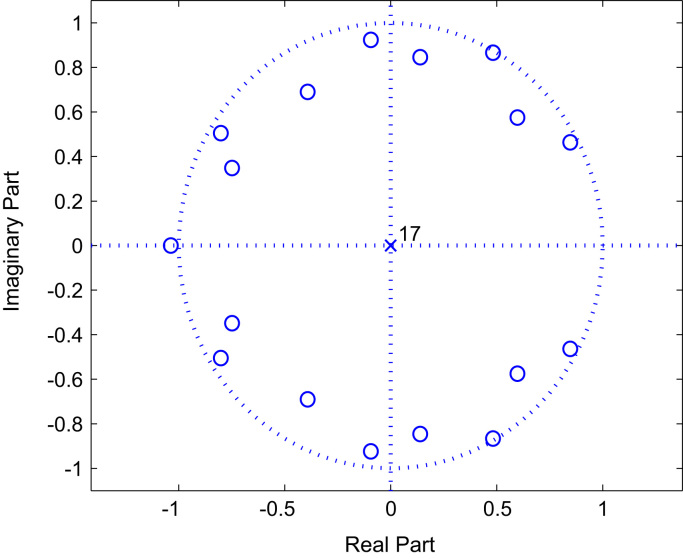
The zeros of Model BRAN E.

The Fig. 2, Fig. 3 and 4 represent the zeros of BRAN A, C and E channels respectively.

### 5.1 Non-linear BRAN channels identification using the proposed method

This subsection presents the numerical simulations results in order to show performance of the proposed algorithm for BRAN data channels identification. Monte Carlo simulations, with 100 runs, have been realized with a non-Gaussian input with N=4800. Performance of the proposed blind non-linear estimator are illustrate in the Table 4, Table 5, Table 6.

The non-linear BRAN A channel, presented in the Table 1, is described by the following model (Eq. 34):

(34) $$\left\{ \begin{array}{c} z(k)=x^{2}(k)+0.9016x^{2}(k-1)+0.8222x^{2}(k-2)+0.7413x^{2}(k-3)+0.6683x^{2}(k-4)\\ +0.6095x^{2}(k-5)+0.5495x^{2}(k-6)+0.4955x^{2}(k-7)+0.4519x^{2}(k-8)+0.4074x^{2}(k-9)\\ +0.5821x^{2}(k-10)+0.4315x^{2}(k-11)+0.3199x^{2}(k-12)+0.2371x^{2}(k-13)+0.2065x^{2}(k-14)\\ +0.1259x^{2}(k-15)+0.0759x^{2}(k-16)+0.0462x^{2}(k-17)\\ \mathrm{Roots}:z_{1}=-0.8604+0.2698i;z_{2}=-0.8604-0.2698i;z_{3}=-0.7103;z_{4}=-0.5926+0.5348i;\\ z_{5}=-0.5926-0.5348i;z_{6}=-0.4801+0.7407i;z_{7}=-0.4801-0.7407i;z_{8}=0.8398+0.3586i;z_{9}=0.8398-0.3586i;\\ z_{10}=0.4491+0.7526i;z_{11}=0.4491-0.7526i;z_{12}=0.5577+0.5615i;z_{13}=0.5577-0.5615i;\\ z_{14}=-0.0340+0.8807i;z_{15}=-0.0340-0.8807i;z_{16}=0.0250+0.7186i;z_{17}=0.0250-0.7186i. \end{array}\right.$$

**Table 1:** Delay and magnitudes of 18 targets of BRAN A channel.

Delay $\tau_{i}[\mathrm{ns}]$	Mag. $A_{i}[\mathrm{dB}]$	Delay $\tau_{i}[\mathrm{ns}]$	Mag. $A_{i}[\mathrm{dB}]$
0	0	90	−7.8
10	−0.9	110	−4.7
20	−1.7	140	−7.3
30	−2.6	170	−9.9
40	−3.5	200	−12.5
50	−4.3	240	−13.7
60	−5.2	290	−18
70	−6.1	340	−22.4
80	−6.9	390	−26.7

The non-linear BRAN C channel, presented in the Table 2, is described by the following model (Eq. 35):

(35) $$\left\{ \begin{array}{c} z(k)=0.6839x^{2}(k)+0.6607x^{2}(k-1)+0.6383x^{2}(k-2)+0.6166x^{2}(k-3)+x^{2}(k-4)\\ +0.9016x^{2}(k-5)+0.8222x^{2}(k-6)+0.7413x^{2}(k-7)+0.8414x^{2}(k-8)+0.7079x^{2}(k-9)\\ +0.6026x^{2}(k-10)+0.5070x^{2}(k-11)+0.5433x^{2}(k-12)+0.4027x^{2}(k-13)+0.3388x^{2}(k-14)\\ +0.2188x^{2}(k-15)+0.1531x^{2}(k-16)+0.0871x^{2}(k-17)\\ \mathrm{Roots}:z_{1}=-0.7190+0.6194i;z_{2}=-0.7190-0.6194i;z_{3}=-0.8576+0.3165i;z_{4}=-0.8576-0.3165i;\\ z_{5}=-0.7080;z_{6}=-0.4573+0.7707i;z_{7}=-0.4573-0.7707i;z_{8}=-0.2292+0.8042i;z_{9}=-0.2292-0.8042i;\\ z_{10}=0.8351+0.4069i;z_{11}=0.8351-0.4069i;z_{12}=0.6756+0.6649i;z_{13}=0.6756-0.6649i;\\ z_{14}=0.2096+0.8143i;z_{15}=0.2096-0.8143i;z_{16}=0.4138+0.7778i;z_{17}=0.4138-0.7778i. \end{array}\right.$$

**Table 2:** Delay and magnitudes of 18 targets of BRAN C channel.

Delay $\tau_{i}[\mathrm{ns}]$	Mag. $A_{i}[\mathrm{dB}]$	Delay $\tau_{i}[\mathrm{ns}]$	Mag. $A_{i}[\mathrm{dB}]$
0	−3.3	230	−3.0
10	−3.6	280	−4.4
20	−3.9	330	−5.9
30	−4.2	400	−5.3
50	0.0	490	−7.9
80	−0.9	600	−9.4
110	−1.7	730	−13.2
140	−2.6	880	−16.3
180	−1.5	1050	−21.2

Also, the non-linear BRAN E channel, presented in the Table 3, is described by the following model (Eq. 36) :

(36) $$\left\{ \begin{array}{c} z(k)=0.5689x^{2}(k)+0.5559x^{2}(k-1)+0.5495x^{2}(k-2)+0.9120x^{2}(k-3)+0.8610x^{2}(k-4)\\ +0.8035x^{2}(k-5)+0.9661x^{2}(k-6)+0.8710x^{2}(k-7)+0.7852x^{2}(k-8)+x^{2}(k-9)\\ +0.8035x^{2}(k-10)+0.7244x^{2}(k-11)+0.5370x^{2}(k-12)+0.4315x^{2}(k-13)+0.2951x^{2}(k-14)\\ +0.2138x^{2}(k-15)+0.1349x^{2}(k-16)+0.0902x^{2}(k-17)\\ \mathrm{Roots}:z_{1}=0.8462+0.4636i;z_{2}=0.8462-0.4636i;z_{3}=0.4826+0.8661i;z_{4}=0.4826-0.8661i;\\ z_{5}=0.5973+0.5751i;z_{6}=0.5973-0.5751i;z_{7}=-1.0372;z_{8}=-0.8011+0.5045i;z_{9}=-0.8011-0.5045i;\\ z_{10}=-0.7480+0.3486i;z_{11}=-0.7480-0.3486i;z_{12}=-0.3919+0.6898i;z_{13}=-0.3919-0.6898i;\\ z_{14}=-0.0939+0.9235i;z_{15}=-0.0939-0.9235i;z_{16}=0.1389+0.8454i;z_{17}=0.1389-0.8454i; \end{array}\right.$$

**Table 3:** Delay and magnitudes of 18 targets of BRAN E channel.

Delay $\tau_{i}[\mathrm{ns}]$	Mag.$A_{i}[\mathrm{dB}]$	Delay $\tau_{i}[\mathrm{ns}]$	Mag.$A_{i}[\mathrm{dB}]$
0	−4.9	320	0.0
10	−5.1	430	−1.9
20	−5.2	560	−2.8
40	−0.8	710	−5.4
70	−1.3	880	−7.3
100	−1.9	1070	−10.6
140	−0.3	1280	−13.4
190	−1.2	1510	−17.4
240	−2.1	1760	−20.9

**Table 4:** Estimation of the BRAN A radio channel impulse response for different SNR and data length N=4800.

h^(i,i)±std	SNR=0dB	SNR=8dB	SNR=16dB	SNR=24dB
h^(0,0)±std	0.9603±0.1342	1.0125±0.0738	1.0157±0.0549	1.0005±0.0415
h^(1,1)±std	0.9575±0.2656	0.9112±0.0603	0.8965±0.0730	0.8879±0.0767
h^(2,2)±std	0.9441±0.2378	0.8377±0.0784	0.8160±0.0931	0.8148±0.0992
h^(3,3)±std	0.7614±0.2229	0.7438±0.0861	0.7133±0.1180	0.7292±0.1186
h^(4,4)±std	0.6350±0.2831	0.6966±0.1113	0.6608±0.1239	0.6796±0.1192
h^(5,5)±std	0.6663±0.3158	0.6489±0.1365	0.5980±0.1286	0.6236±0.1070
h^(6,6)±std	0.5633±0.0988	0.5591±0.0499	0.5594±0.0619	0.5623±0.0475
h^(7,7)±std	0.5405±0.0717	0.5009±0.0506	0.4965±0.0647	0.4860±0.0464
h^(8,8)±std	0.5328±0.0915	0.4706±0.0728	0.4627±0.0820	0.4524±0.0540
h^(9,9)±std	0.4397±0.0996	0.4199±0.0645	0.4141±0.0766	0.3958±0.0473
h^(10,10)±std	0.6163±0.0768	0.6028±0.0430	0.6021±0.0623	0.5967±0.0465
h^(11,11)±std	0.5293±0.1000	0.4413±0.0720	0.4423±0.0870	0.4321±0.0479
h^(12,12)±std	0.2430±0.2209	0.3080±0.0456	0.3402±0.0863	0.3298±0.0659
h^(13,13)±std	0.2349±0.2501	0.2476±0.0580	0.2226±0.0754	0.2320±0.0660
h^(14,14)±std	0.2113±0.5071	0.2511±0.0957	0.2282±0.1403	0.2310±0.1030
h^(15,15)±std	0.1549±0.3954	0.1581±0.0758	0.1406±0.1197	0.1626±0.1019
h^(16,16)±std	0.1816±0.3102	0.1511±0.1293	0.0970±0.0920	0.1005±0.1013
h^(17,17)±std	0.0796±0.5222	0.0392±0.1667	0.0116±0.2398	0.0399±0.2689
MSE	0.1508	0.0629	0.0375	0.0127

### 5.2 BER performance of MC-CDMA systems

In this subsection we present numerical simulation results of the measured and estimated BER in MC-CDMA systems . These estimation are preformed using MMSE and ZF equalizers.

**Table 5:** Estimation of the BRAN C radio channel impulse response for different SNR and data length N=4800.

h^(i,i)±std	SNR=0dB	SNR=8dB	SNR=16dB	SNR=24dB
h^(0,0)±std	0.7398±0.1054	0.7272±0.0837	0.7196±0.0562	0.7154±0.0751
h^(1,1)±std	0.7930±0.1759	0.7162±0.1072	0.6672±0.0870	0.6682±0.0984
h^(2,2)±std	0.8880±0.2260	0.7079±0.1094	0.6562±0.0998	0.6600±0.1193
h^(3,3)±std	0.7833±0.2484	0.6495±0.1405	0.6185±0.0941	0.6148±0.1013
h^(4,4)±std	1.0723±0.3335	1.0165±0.1841	1.0031±0.1586	1.0032±0.1416
h^(5,5)±std	1.0909±0.3443	0.9365±0.1435	0.9094±0.1308	0.9037±0.1225
h^(6,6)±std	0.7813±0.1094	0.8351±0.0578	0.8407±0.0459	0.8383±0.0733
h^(7,7)±std	0.7219±0.1134	0.7491±0.0744	0.7402±0.0722	0.7427±0.0982
h^(8,8)±std	0.8900±0.1529	0.8391±0.0849	0.8392±0.0924	0.8315±0.0867
h^(9,9)±std	0.6925±0.1747	0.6760±0.0791	0.6972±0.0957	0.6990±0.1094
h^(10,10)±std	0.5878±0.1228	0.5846±0.1049	0.6027±0.0969	0.6042±0.1153
h^(11,11)±std	0.5902±0.1722	0.5118±0.1005	0.4976±0.0941	0.5046±0.1215
h^(12,12)±std	0.5201±0.0799	0.5207±0.0858	0.5548±0.0473	0.5562±0.0453
h^(13,13)±std	0.4026±0.0710	0.3986±0.0837	0.3918±0.0701	0.4101±0.0759
h^(14,14)±std	0.4251±0.1317	0.3996±0.1386	0.3706±0.1607	0.3374±0.0730
h^(15,15)±std	0.2761±0.1558	0.2662±0.1199	0.2253±0.1348	0.2304±0.1134
h^(16,16)±std	0.1883±0.1170	0.1806±0.1526	0.2030±0.1609	0.2049±0.1300
h^(17,17)±std	0.1298±0.1916	0.1050±0.2373	0.0953±0.2487	0.0935±0.1606
MSE	0.0436	0.0104	0.0073	0.0071

**Table 6:** Estimation of the BRAN E radio channel impulse response for different SNR and data length N=4800.

h^(i,i)±std	SNR=0dB	SNR=8dB	SNR=16dB	SNR=24dB
h^(0,0)±std	0.5914±0.1750	0.6155±0.0713	0.5944±0.0931	0.5938±0.0763
h^(1,1)±std	0.6892±0.1594	0.5882±0.0815	0.5552±0.0798	0.5481±0.0762
h^(2,2)±std	0.7438±0.1917	0.5731±0.1236	0.5441±0.0696	0.5438±0.1015
h^(3,3)±std	1.1086±0.3365	0.9006±0.1120	0.8880±0.1090	0.8971±0.1561
h^(4,4)±std	1.0241±0.3077	0.8846±0.1134	0.8710±0.0992	0.8623±0.1459
h^(5,5)±std	1.0118±0.2844	0.8276±0.1023	0.8232±0.0879	0.8098±0.1120
h^(6,6)±std	1.0184±0.2365	0.9870±0.0845	0.9933±0.0815	0.9756±0.0518
h^(7,7)±std	0.9175±0.2056	0.9153±0.1136	0.8677±0.0866	0.8697±0.0655
h^(8,8)±std	1.0146±0.3212	0.8339±0.1650	0.7719±0.1234	0.7807±0.1051
h^(9,9)±std	0.9804±0.3522	0.9888±0.1812	0.9407±0.1938	0.9968±0.1337
h^(10,10)±std	0.8474±0.2253	0.8339±0.1588	0.7873±0.1638	0.8075±0.1193
h^(11,11)±std	0.8303±0.3533	0.7834±0.1787	0.7014±0.1398	0.7525±0.1478
h^(12,12)±std	0.4960±0.0992	0.5145±0.0889	0.5350±0.0511	0.5349±0.0373
h^(13,13)±std	0.4497±0.0788	0.4048±0.0936	0.4182±0.0475	0.3925±0.0524
h^(14,14)±std	0.3794±0.1307	0.3118±0.1272	0.2816±0.0590	0.2641±0.0811
h^(15,15)±std	0.2764±0.1221	0.2209±0.1019	0.2076±0.1002	0.1862±0.0846
h^(16,16)±std	0.1629±0.1403	0.1364±0.1265	0.1574±0.1243	0.1573±0.1299
h^(17,17)±std	0.0966±0.1851	0.0592±0.2331	0.1140±0.1803	0.0932±0.1867
MSE	0.0374	0.0087	0.0062	0.0038

Performances quality is evaluated under the following conditions (Table 7):

**Table 7:** The simulation parameters.

Simulation parameters	Values
Number of BPSK symbols	$2^{14}$
Size of the FFT	64
Spreading code	Walsh-Hadamard
Length of the spreading code	64
Number of users	64
Channel type	BRAN: A/ C/ E
MonteCarlo runs used to compute the estimated BER	5
Data from the user to detect	1

In the Table 8, Table 9, Table 10 we represents the real and estimated BER of the BRAN A, C and E channels respectively, using ZF and MMSE equalizers.

**Table 8:** Real and estimated BER for various SNR using ZF and MMSE equalizers in BRAN A channel.

SNR (dB)	Real BER: ZF	Estimated BER: ZF	Real BER: MMSE	Real BER: MMSE
0	0.3421	0.4610	0.2333	0.2431
4	0.2561	0.3019	0.1502	0.1531
8	0.1495	0.2317	0.0641	0.0670
12	0.0504	0.0556	0.0113	0.0120
16	0.0038	0.0050	0.0004	0.0004
20	0.00003	0.00002	0.00003	0.00003
24	0.00003	0.00002	0.00002	0.00002
28	0.00003	0.00002	0.00002	0.00002

**Table 9:** Real and estimated BER for various SNR using ZF and MMSE equalizers in BRAN C channel.

SNR (dB)	Real BER: ZF	Real BER: ZF	Real BER: MMSE	Real BER: MMSE
0	0.4313	0.4959	0.2657	0.2757
4	0.3910	0.4962	0.1973	0.2015
8	0.3292	0.4235	0.1079	0.1083
12	0.2409	0.3019	0.0363	0.0369
16	0.1058	0.1359	0.0048	0.0053
20	0.0396	0.0593	0.0002	0.0003
24	0.0029	0.0080	0.00003	0.00003
28	0.00008	0.0002	0.00003	0.00003

**Table 10:** Real and estimated BER for various SNR using ZF and MMSE equalizers in BRAN E channel.

SNR (dB)	Real BER: ZF	Estimated BER: ZF	Real BER: MMSE	Estimated BER: MMSE
0	0.4135	0.4331	0.2795	0.2948
4	0.3959	0.4879	0.2099	0.2159
8	0.3248	0.3347	0.1231	0.1276
12	0.2199	0.2518	0.0442	0.0451
16	0.1075	0.1463	0.0067	0.0078
20	0.0334	0.0465	0.0002	0.0002
24	0.0043	0.0065	0.00002	0.00004
28	0.0002	0.0010	0.00002	0.00004

## 6 Concluding remarks

In this paper we have proposed a blind non-linear approach, based on fourth order cumulants, for estimation of the non-linear BRAN channels excited by non-Gaussian and independent identically distributed signal. According to the analytic study and numerical simulations results we can draw the following remarks:

• The proposed estimator able to estimate the diagonal parameters of the quadratic BRAN channels from the output signal without knowing the system input i.e. blindly;
• The developed approach is based on simple equations that we have used only (q+1) equations to estimate *q* parameters;
• The measured values of the BRAN channels are very close to the estimated in different SNR, same in very noise environment SNR =0 dB;
• Not affected by the presence of Gaussian noise, because it is vanish in the higher order cumulants domain;
• Gives a good result for a standard deviation.

In the part of blind equalization problem, we consider the 4G MC-CDMA systems. However, we use two equalizers, ZF and MMSE, after the channel identification to correct the channel distortion. According BER simulation results we can conclude the following:

• Using ZF equalizer, the BER simulation for various SNR between 0 dB and 28 dB, demonstrates that the estimated values are more close to the real value of all data BRAN, and we have a best accorded;
• Using MMSE equalizer, the obtained results show a prefect accorded between the real and estimated BER. Indeed, for example using BRAN A data, if the SNR ≥ 20 dB we have 1 bit error if we receive $10^{5}$ bit.

Finally, the proposed estimator combining with the MMSE equalizer can compensate the distortion introduite by radio channel in noise environment.

## Appendix A Supporting Information

Application 1
