# Supplementary material for: Measured and estimated data of non-linear BRAN channels using HOS in 4G wireless communications
Source: Data Brief. 2018 Feb 10;17:1136–48. doi: 10.1016/j.dib.2018.02.005 (PMC5988528; doi:10.1016/j.dib.2018.02.005)
Supplement: Application 1 [file mmc1.pdf]

Conflict of interests

The authors declare that there is no conflict of interest regarding the publication of this paper.
